# New York Physicians' Perspectives and Knowledge of the State Medical Marijuana Program

**DOI:** 10.1089/can.2017.0046

**Published:** 2018-03-01

**Authors:** Alexandra Sideris, Fahad Khan, Alina Boltunova, Germaine Cuff, Christopher Gharibo, Lisa V. Doan

**Affiliations:** Department of Anesthesiology, Perioperative Care and Pain Medicine, New York University Langone Health, New York, New York.

**Keywords:** medical marijuana, New York, opioids, pain, physicians, survey

## Abstract

**Introduction:** In 2014, New York (NY) became the 23rd state to legalize medical marijuana (MMJ). The purpose of this survey was to collect data about practicing NY physicians' comfort level, opinions, and experience in recommending or supporting patient use of MMJ.

**Materials and Methods:** An anonymous web-based survey was distributed to medical societies and to academic departments in medical schools within NY.

**Results:** A total of 164 responses were analyzed. Physician participants were primarily located in New York City and surrounding areas. The majority (71%) agreed that MMJ should be an option available to patients. Most respondents were not registered to certify MMJ in NY, but were willing to refer patients to registered physicians. Common reasons for not registering included specialty and federal status of cannabis. More than 75% reported having patients who used cannabis for symptom control, and 50% reported having patients who inquired about MMJ within the past year. Most respondents are willing to discuss MMJ with their patients, but had little familiarity with the state program and a modest knowledge of the endocannabinoid system. Pain was a common symptom for which cannabis was recommended by registered physicians (69%) and purportedly used by patients (83%). Most respondents would consider MMJ as an adjuvant to opioids, and 84% believed opioids have greater risks than MMJ.

**Conclusion:** Given that the majority of surveyed physicians support MMJ as an option for patients, few are registered and have adequate knowledge of MMJ. Although our study sample is small and geographically limited, our survey results highlight key physician issues that are likely applicable to practitioners in other states. Concerted efforts are needed at the federal, state, and academic levels to provide practitioners with evidence-based guidelines for the safe use of MMJ.

## Introduction

On July 7th, 2014, the Compassionate Care Act was signed into law, making New York (NY) the 23rd state to legalize medical marijuana (MMJ).^1^ As of November 2017, 29 states and Washington, DC, have passed MMJ laws, 17 additional states passed cannabidiol (CBD)-specific laws, and 8 states and DC legalized recreational cannabis.^2,3^ In contrast, the federal government currently prohibits the use of botanical cannabis and its constituents for medical purposes. They are classified as Schedule I under the Controlled Substances Act—substances considered to be the most harmful and conferring no medical benefits.^4^ With the exception of three Food and Drug Administration (FDA)-approved synthetic cannabinoid drugs, botanical cannabis and its constituents cannot be prescribed or legally dispensed outside of a federally approved research program. Physicians residing in states with MMJ laws, including NY, can only “certify” that patients have a qualifying condition and may “recommend” MMJ use, but cannot issue a prescription.^5,6^

In the original law, the New York State Medical Marijuana Program (NY-MMP) had strict regulations for patients, providers, caregivers and dispensaries.^2,7,8^ Only 10 medical conditions were approved and a patient had to present with at least one of five qualifying symptoms. The qualifying conditions were as follows: amyotrophic lateral sclerosis, cancer, epilepsy, HIV/AIDS, Huntington's disease, inflammatory bowel disease, multiple sclerosis, neuropathy, Parkinson's disease, and spinal cord injury with spasticity. At least one of the following qualifying symptoms must have been present with a qualifying condition: severe or chronic pain, severe or persistent muscle spasms, seizures, cachexia, or nausea.^8^ Physicians who registered to certify patients must complete a 4-h course that provides an overview of the endocannabinoid (eCB) system, physiological and adverse effects of cannabinoids, and guidelines for dosing and administration. The NY-MMP combined several safeguards to mitigate potential diversion and MMJ abuse, and to ensure product safety.^9,10^ No smoking or possession of raw plant material was allowed; only oils, liquids, or pills that were either ingested or vaporized were permitted. There was a 30-day supply limit, no individual dose contained >10 mg of Δ^9^-tetrahydrocannabinol (THC), the major psychoactive compound found in cannabis, and dispensaries are required to report data to the Prescription Drug Monitoring Program Registry (PDMP). NY has since amended these laws to include two new qualifying conditions, to allow both nurse practitioners and physician assistants to register as practitioners, to reduce the educational requirement from 4 to 2 hours, and to expand the types of MMJ available to patients.^11^

Physicians began registering with the NY-MMP in October 2015, and began certifying patients on December 23rd, 2015. Patients began obtaining MMJ on January 7th, 2016.^12^ To date, no physician survey about the NY-MMP has been published. The purpose of this survey was to probe NY physicians' knowledge and perspectives of the MMP and cannabinoids in patient care. As a large proportion of patients in other states with MMPs are purportedly certified to use MMJ for chronic or severe pain,^13–15^ there was a particular interest to assess physicians' opinions regarding MMJ for the management of pain.

## Methods

This study was reviewed and approved by the New York University School of Medicine Institutional Review Board.

### Instrument

A 30-item questionnaire was developed by our research team based on surveys developed by others,^16,17^ but adapted to NY-specific needs (Supplementary Data—Survey). This survey sought to obtain perspectives on the NY-MMP from practicing physicians. Survey questions included categorical/nominal, ordinal, and continuous data. To safeguard potentially sensitive information, participants were given the option to choose “Prefer not to answer” in several questions. Importantly, completion of the survey in its entirety was voluntary.

### Study participants

Participants were practicing physicians, MDs or DOs, in NY. Survey responses not meeting these criteria were excluded from the analysis. Physicians were not compensated for their participation.

### Recruitment

Professional NY medical organizations and county and specialty society officers from the Medical Society of NY were contacted. The survey request was also sent to the New York City (NYC) Health Commissioner, to the organization “Compassionate Care NY,” and to department chairs in academic medical centers in NY to disseminate to their faculty members. A total of 37 medical specialty societies, 60 county societies of the Medical Society of NY, and 12 academic medical centers were contacted across NY to participate in the study (Supplementary Table S1).

### Data collection

Study data were collected and stored in a password-protected account using the online survey service company Survey Monkey. To further protect anonymity of participants to stop IP address tracking, “Anonymous Responses” was turned on in the survey settings. Only the principal investigator and actively involved researchers had access to the survey results. Strict control of data was maintained and no personal identifiers were collected or reported in the findings. Data were collected between August 3rd, 2016, and July 11th, 2017.

### Analysis

Survey responses from close-ended questions were sorted and analyzed using Survey Monkey and Excel, and Prism statistical software. Responses from open-ended questions were analyzed through thematic categorization and frequency analysis.

## Results

### Demographics

A total of 4 medical specialty societies, 11 county societies, and 6 academic departments from 3 medical schools agreed to participate in the study. All specialty societies had state-wide members; two were related to the pain specialty, one was anesthesiology, and the other was a family medicine society. Of the county societies that agreed to participate, one was a borough in NYC, three were surrounding counties, and seven counties were from Upstate. Of the medical schools that participated, one was located in Upstate NY and the others in NYC. The six academic departments were anesthesiology (2), physical medicine and rehabilitation (1), psychiatry (1), ophthalmology (1), and oncology (1).

**FIG. 1.**
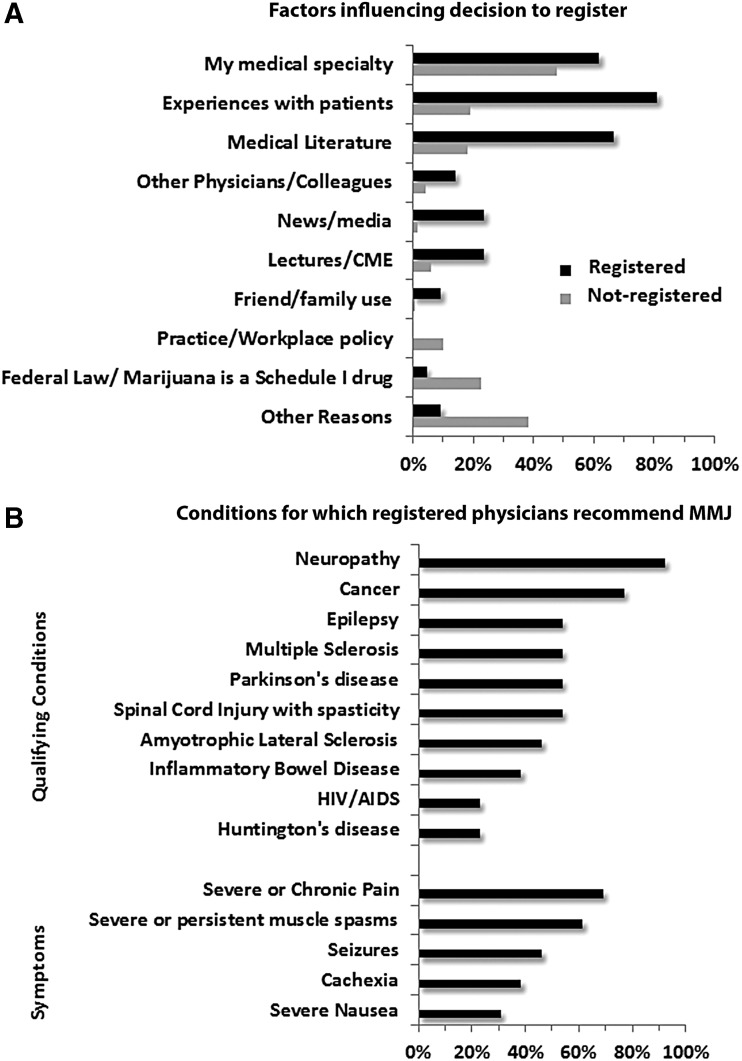
Factors influencing decision to register and conditions for which registered physicians recommend MMJ. **(A)** Registered (black) and not registered (gray) physician participants (*n*=133 answered) were given the option to select more than one factor, and/or list other reasons. **(B)** Qualifying conditions and associated symptoms for which registered physicians currently or anticipate recommending MMJ (*n*=13 answered). CME, continuing medical education; MMJ, medical marijuana.

A total of 167 respondents participated in the survey. Three participants did not meet inclusion criteria. Most participants ranged in age between 45 and 64 (52%), and were in practice for over 25 years (37%) (Table 1). Of these participants, 86% held MD degrees only, 10% (17/164) held DO degrees, and 4% (6/164) held more than one advanced graduate degree (5 MD/PhD and 1 MD/MBA). A total of 152 respondents reported their specialty; the five most prevalent reported specialties were primary care (19%), anesthesiology (18%), pain medicine (15%), surgery (14%), and psychiatry (9%). Overall, 87% (*n*=142) of respondents were not registered; only 8% were planning to register within the next year, but 73% were willing to recommend patients to registered physicians. Twenty-one (∼13%) of the respondents were registered with the MMP. Of these, 85% were in primary care, pain medicine, or palliative medicine and 75% were in private practice. Nonregistered physicians fell equally into academic/university and private practice settings (Table 1). Medical specialty and federal status of cannabis were primary reasons for not registering (Fig. 1A). Respondents in academic medical centers were more likely than those in private practice to indicate federal status of cannabis (26% vs. 14%) and workplace policy (15% vs. 4%) as reasons influencing their decision not to register. Other factors for not registering included experiences with patients, medical literature, not enough evidence on the efficacy or safety of MMJ, a burdensome registration process, and lack of knowledge of the MMP and/or MMJ (Supplementary Table S2).

**Table 1.** **Demographics of Survey Respondents**

	All respondents		Registered		Not registered	
	*n*	%	*n*	%	*n*	%
Specialty						
Primary care	29	19.2	9	45	20	15.3
Pain medicine	23	15.2	6	30	17	13.0
Palliative medicine	3	2.0	2	10	1	0.8
Anesthesiology	28	18.5	1	5	27	20.6
Surgical specialties	21	13.9	—	—	21	16.0
Psychiatry	14	9.3	1	5	13	9.9
Oncology	8	5.3	—	—	8	6.1
PMR	7	4.7	—	—	7	5.3
Other^*^	18	11.9	1	5	17	13.0
Practice setting						
Academic	63	39.6	2	10	63	46.7
Private	72	45.3	15	75	57	42.2
Public hospital (non-VA)	12	7.5	—	—	12	8.9
Other^#^	12	7.5	4	20	3	2.2
Gender						
Male	102	62.6	13	61.9	89	63.1
Female	60	36.8	8	38.1	52	36.6
Other	1	0.6	—	—	1	0.7
Age						
25–34	22	13.4	2	9.5	20	14.0
35–44	31	18.9	—	—	31	21.7
45–54	43	26.2	8	38.1	35	24.5
55–64	42	25.6	7	33.3	35	24.5
65–74	22	13.4	4	19	18	12.6
>75	4	2.4	—	—	4	2.8
Years in practice						
<1–5	33	20.1	2	9.5	31	21.7
6–10	20	12.2	—	—	20	14.0
11–15	15	9.1	1	4.8	14	9.8
16–20	18	11.0	3	14.3	15	10.5
21–25	17	10.4	3	14.3	14	9.8
>25	61	37.2	12	57.1	49	34.3
Location						
NYC	60	43.2	5	26.3	55	45.8
Westchester	28	20.1	6	31.6	22	18.3
Long Island	14	10.1	1	5.26	13	10.8
Upstate	37	26.6	7	36.8	30	25.0

Percentages are calculated from the total number of participants who answered the question per category. Specialty (*n*=151); primary care includes internal medicine (*n*=12) and family medicine (*n*=17); surgery, surgical specialties: orthopedic surgery (*n*=5); neurosurgery (*n*=3); urology (*n*=3); ophthalmology (*n*=3); OB/GYN (*n*=2); breast (*n*=1); thoracic (*n*=1); and general (*n*=1).

^*^ Other specialties (*n*=1 for each): critical care, pediatrics, cardiology, dermatology, neurology, emergency medicine, occupational medicine, geriatrics, endocrinology, and functional medicine. For practice setting (*n*=159); ^#^Other practice settings included hospital-owned clinic, ambulatory practice salaried with regional healthcare system, nursing home-free clinic, nonprofit hospice organization, rural clinic, college health service, former academic-now private practice, and hospice/nonprofit; gender (*n*=163); age and years in practice (*n*=164); location/county (*n*=139): NYC/Manhattan (*n*=56), Bronx (*n*=2), Queens (*n*=1), and Richmond (*n*=1); Long Island/Suffolk (*n*=13) and Nassau (*n*=1); Upstate/Tompkins (*n*=9), Broome (*n*=8), Otsego (*n*=4), Onondaga (*n*=3), Erie (*n*=2), Schoharie (*n*=2), and *n*=1 from Cattaraugus, Chemung, Delaware, Dutchess, Oneida, Lewis, Tioga, Rensselaer, and Ulster.

NYC, New York City; OB/GYN, obstetrics and gynecology; PMR, physical medicine and rehabilitation; VA, Veterans Affairs.

Among registered physicians, neuropathy (93%) and cancer (77%) were the most common conditions, while pain (69%) was the most common symptom for which MMJ was recommended (Fig. 1B). With regard to the state-mandated education course, the percentage that agreed (42%) with the statement that the New York State Department of Health education course provided sufficient information about MMJ was similar to those that disagreed (47%).

### Assessment of physicians' knowledge and perspectives of MMJ and NY Program

**FIG. 2.**
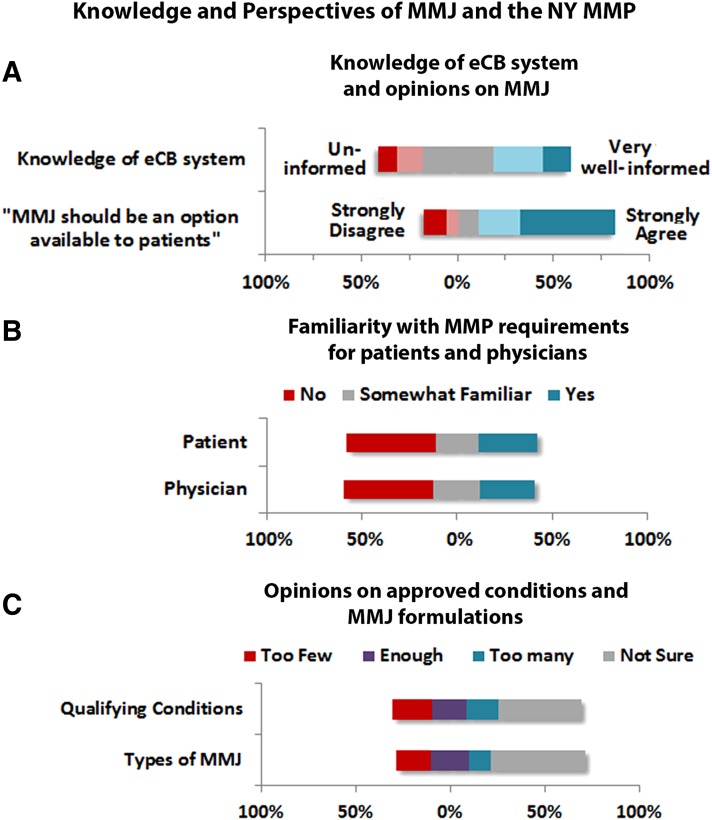
Knowledge and perspectives of MMJ and the NY-MMP. Participants (*n*=164 answered) rated **(A)** their knowledge of the eCB system, the extent to which they agree with the statement that MMJ should be an option available to patients, **(B)** their familiarity with the patient (*n*=163 answered) and physician requirements for participating in the MMP, and **(C)** their opinions regarding the number of qualifying conditions and available MMJ formulations. eCB, endocannabinoid; NY-MMP, New York State Medical Marijuana Program.

When participants were asked to rate their knowledge of the eCB system on a 5-point rating scale (1, uninformed, and 5, very well informed), 60% were somewhat or not well-informed (1–3) and 26% were informed (4), but only 14% were very well informed (5). The majority of respondents (71.2%) believed that MMJ should be an option available to patients (Fig. 2A). Regarding knowledge of the NY-MMP, overall, 45% and 47% of respondents reported no familiarity with the requirements for patients and physicians, respectively, to participate (Fig. 2B). The greatest proportion of respondents reported “I am not sure” for both the number of qualifying conditions (44%) and available formulations (50%) (Fig. 2C). Of the respondents who were registered, 62% (13/21) felt that there were too few qualifying conditions approved in NY.

### Physicians' experience with cannabinoids in patient care

Approximately 95% of registered physicians and 49% of nonregistered physicians had patients who inquired about MMJ within the past year. All pain physicians (23/23) and almost all primary care physicians who answered this question (23/25) had patients who inquired. Most respondents (55%; 86/155) reported a willingness to discuss MMJ with patients, regardless of their registration status or specialty, if they felt their patient could benefit. Greater than 75% (118/156) reported having patients who used cannabis for symptom control: pain (83%; *n*=88), anxiety (54.7%; *n*=58), nausea (46.2%; *n*=49), depression (37.1%; *n*=39), cachexia (31.1%; *n*=33), and 17.9% (*n*=19) for other indications, including for spasticity, sleep issues, and seizures.

Approximately 25% of respondents (39/155) previously prescribed the FDA-approved cannabinoids. While 44% (*n*=17) reported prescribing them strictly for the FDA-approved conditions (nausea and vomiting, and/or appetite stimulation), 54% (*n*=21) reported prescribing them for pain as well. When asked to consider cannabinoids in a patient's treatment regimen, overall, 32.9% (48/146) would first opt for FDA-approved cannabinoids, while 30% (43/146) would choose NY MMJ as a first option. The other 38% (55/146) reported that choosing cannabinoids in their treatment regimen is not applicable to them.

### Perspectives on opioid and cannabis use

**FIG. 3.**
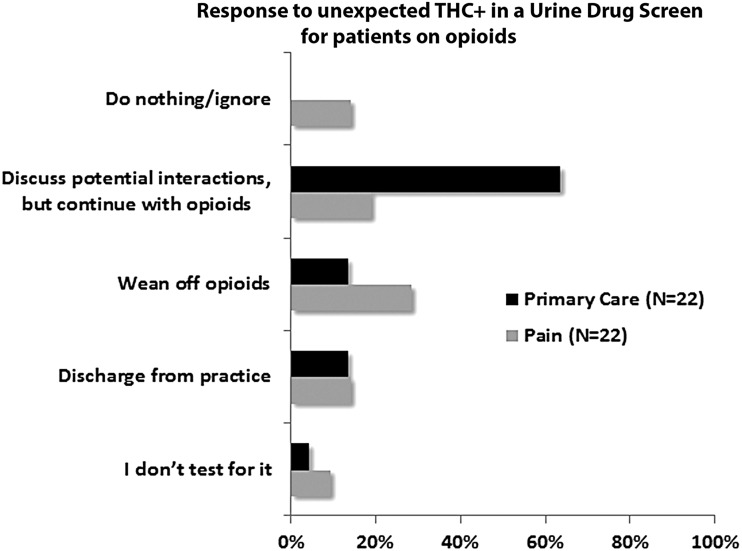
Response to unexpected THC+ in a UDS for patients on opioids. The course of action of primary care and pain medicine physicians, if for an otherwise expected UDS, THC is detected while their patients are using opioids. THC, tetrahydrocannabinol; UDS, urine drug screen.

Most respondents (75%; 116/156) prescribe opioids for their patients; 27% of the opioid prescribers would not consider MMJ as an adjuvant to opioids, 25% would consider it only for cancer pain or palliative care, and 48% would consider adjuvant MMJ for both nonmalignant and cancer pain. Compared to primary care physicians, pain physicians were less likely to consider MMJ as an adjuvant to opioids for both nonmalignant and cancer pain (56% vs. 35%), and were less likely to continue opioid therapy if, for an otherwise expected urine drug screen (UDS), THC was detected (63% vs. 19%) (Fig. 3). Overall, 84% (127/152) believed opioids had greater risks than cannabis, 4% (6/152) believed cannabis had more risks than opioids, and 12% (19/152) were not sure which of the two had more risks.

## Discussion

Despite the fact that more than half of the United States has MMJ laws, little is known about physicians' attitudes toward these programs or their experiences with them. This survey study assessed the experience of practicing NY physicians with the state's MMP. Although the response rate was low, the survey results provide insight into future MMJ educational and research needs. MMJ is emerging as an option for many patients across the country, and there is an increasing trend for its demand.^18^ A May 2014 poll indicated that 88% of NY registered voters supported legalizing MMJ.^19^ Greater than 70% of physicians who took this survey believed that MMJ should be an option available to patients. Many have patients who inquired about or already used cannabis for symptom control; however, the respondents' familiarity with the NY program and the eCB system was modest. Evidence-based guidelines for practitioners are lacking. Given the increasing trend in MMJ use, research to address the safety, tolerability, and efficacy of various cannabis formulations alone and with other medications is an urgent public health need.

**FIG. 4.**
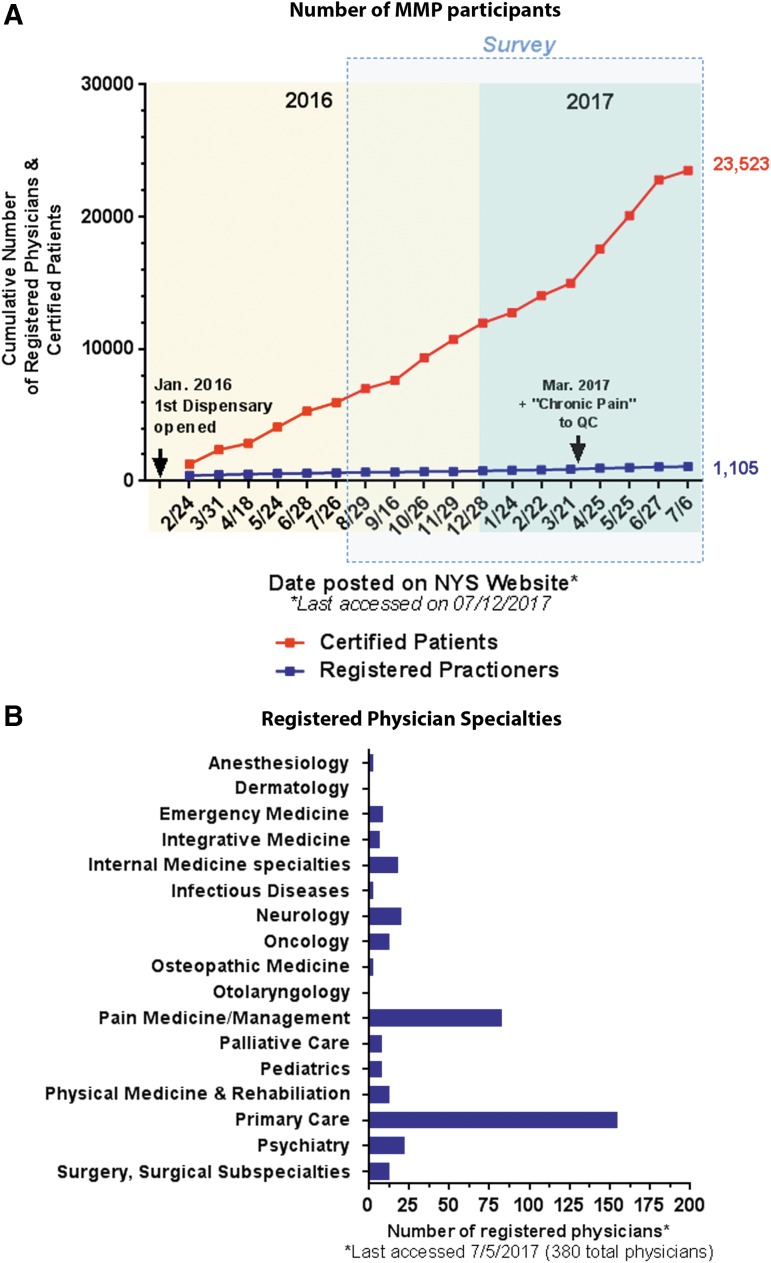
Certified patients and registered physicians in NY, as of July 12th, 2017. **(A)** Cumulative number of certified patients (red) and registered physicians (blue). Data were compiled from the periodic updates posted on the NY-MMP website.^1^ Arrows indicate key events or changes in the MMP. **(B)** Specialties of registered practitioners who opted in to publicly display their contact information on the NY-MMP website^21^ as of July 2017. Note that approximately one-third (380/1105) of the registered practitioners chose to do so. Of these, 41% (155/380) report primary care and 22% (83/380) report pain medicine/management as their specialty.

In NY, during the first 6 months of the program, 4998 patients were certified.^12^ The most common qualifying condition was neuropathy (34.09%), and the most common associated condition or complication was severe or chronic pain (53.53%).^12^ These statistics align with the survey responses of the registered physicians regarding the reasons for certifying patients. As of July 2017, the number of certified patients in the MMP exceeded 20,000.^1^ With the addition of “chronic pain” as an approved qualifying condition in March 2017, ∼7500 new patients were certified by the end of June—a 50% increase in 3 months (Fig. 4).^20^ While patient demand for MMJ is increasing, the growth of registered practitioners in NY is relatively stagnant (Fig. 4A). Most registered physicians in the survey are primary care physicians or pain specialists, and are in private practice. The large discrepancy of registered physicians among different practice settings may be due to several factors, and consequently there may be barriers to MMJ access. The low proportion of registered survey respondents and their reported specialties reflect the practitioner data publicly disclosed by the MMP^21^ (Fig. 4B). While most registered respondents recommended MMJ for pain, there is a dearth of well-controlled studies on the efficacy of MMJ or selective cannabinoids for the management of pain.^22–24^ Furthermore, no studies have been conducted with the cannabinoid formulations available in NY, making it difficult to guide patients on their safe and efficacious use.

During our study, the MMP only allowed the production and dispensing of mixed cannabinoid formulations (THC and CBD) as capsules or as liquid or oil preparations intended for metered oromucosal, sublingual administration or vaporization.^8^ While the FDA has not yet approved any product containing extracts of cannabis, three synthetic cannabinoid medications are approved: dronabinol, available as a capsule or liquid, and nabilone. Dronabinol is FDA approved for appetite stimulation in AIDS patients and for chemotherapy-induced nausea and vomiting (CINV), while nabilone is approved for CINV.^25–27^ Of the survey respondents who prescribed FDA-approved cannabinoids, >50% indicated doing so, in part, for the management of pain. When given the scenario of choosing between FDA-approved cannabinoids or NY MMJ formulations as a first option in a patient's treatment plan, respondents were equally divided between the two. It is not clear why respondents would opt for one over the other, but it is intriguing that FDA-approved cannabinoids are not necessarily favored.

The current opioid epidemic necessitates alternatives for pain control. Associations between the presence of state MMJ laws and decreases in opioid overdose deaths have been noted.^15^ Some patients substitute cannabis for opioids.^28–34^ Evidence suggests that cannabinoids may be effective adjuvants and can affect tolerance to opioids.^35–42^ Although cannabis does not lead to overdose deaths, its use can lead to cannabis use disorder and cognitive impairments,^43,44^ which could lead to risky behavior.^45–50^ Importantly, there is limited information on the risks associated with the use of MMJ alone or in combination with opioids. To our knowledge, this is the first physician survey to assess attitudes toward opioids and MMJ for the management of pain. Most physicians surveyed believe MMJ is safer than opioids. Approximately 75% of the opioid-prescribing respondents would consider recommending adjuvant MMJ, and almost half would do so for either malignant or non-cancer pain. Even if THC was found in their patients' UDS, most primary care respondents would continue with opioid therapy; pain physicians were divided in their approaches. In the 2016 *Pain Treatment Guidelines* from Oregon, another MMJ state, providers may ask their patients to choose between opioids or MMJ, adopt a “don't ask, don't tell” policy, or recommend CBD-rich MMJ as an adjuvant to opioids.^51^ In the CDC *Guideline for Prescribing Opioids for Chronic Pain*, UDS for THC is not advocated, stating that the clinical implications of doing so are not clear.^52^ Our survey results reflect that there is no consensus for how to manage opioid and cannabis use, underscoring the crucial importance of addressing their concurrent use in pain management.^53^

Physician surveys on cannabis have been conducted in several states and nationwide.^16,17,54–57^ Although MMJ state laws vary widely, common concerns among healthcare practitioners are legal uncertainties, inadequate knowledge, questionable efficacy, and abuse potential of cannabis. At the base of these concerns is the Schedule I status of cannabis, which creates legal ambiguity for recommending practitioners and imposes regulatory burden in the study of cannabis and its extracts. While the federal government supports therapeutic cannabinoid research and provides cannabis plant material,^58^ the current cannabis strains available from the National Institute on Drug Abuse do not reflect what is available in state legal markets.^59^ Well-controlled studies with cannabis products used in state MMPs are needed to address research gaps. With regard to abuse potential, recent national data indicate that up to 30% of cannabis users may develop cannabis use disorder.^43^ Furthermore, numerous reports link cannabis use with exacerbated or emergent psychotic symptoms, particularly in patients with a predisposition.^50^ Additional research is needed to harness the therapeutic potential of cannabis and to minimize potential risks to individuals.

## Limitations of the Study

There are several limitations in this study. The response rate was low, not all specialties were represented, and respondents were primarily located in two NY counties. Moreover, we cannot rule out the possibility of self-selection bias of survey participants. Societies and individuals who decided to participate in the study may already hold strong viewpoints regarding MMJ in patient care. Thus, the findings of the study may not be generalizable to all NY physicians. Furthermore, after the survey was disseminated, the MMP was expanded to allow nurse practitioners and physician assistants to register, and approved two more qualifying conditions. As the MMP evolves, future surveys to assess the perspectives of a broader group of practitioners may lead to a better understanding of education and policy needs.

## Future Directions

In accordance with recommendations made in the state's 2-year report, NY continues to enhance its MMP to provide additional educational resources and streamline the registration process.^12,60,61^ This survey study emphasizes that concerted efforts are needed to address the safety and efficacy of cannabinoids, particularly in pain management, so that practitioners are better able to inform and care for their patients.

From a patient's perspective, it is not clear if cannabis helps control symptoms and if state certification is a desirable option. Future surveys to understand cannabis use from patients' perspectives are needed. NY, Connecticut, and Ohio are the only MMJ states requiring dispensaries to report data to their PDMP registries.^11,62,63^ These states are uniquely poised to elucidate opioid-MMJ dispensing patterns. Interrogations of these patterns should yield novel insights into practitioners' regimens, patients' needs, and health outcomes at the state level.

Policies regarding recreational and MMJ continue to change rapidly. The potential future sanctioned use of MMJ from a federal perspective will depend on a more complex interplay between several stakeholders, including the FDA, Drug Enforcement Administration, and the National Institutes of Health. Ultimately, more research is needed to drive policy and guide the safe use of cannabinoids.

## Supplementary Material

Supplemental data

Supplemental data

Supplemental data

## Abbreviations Used

CBD: cannabidiol
CINV: chemotherapy-induced nausea and vomiting
CME: continuing medical education
eCB: endocannabinoid
FDA: Food and Drug Administration
MMJ: medical marijuana
NY: New York
NYC: New York City
NY-MMP: New York State Medical Marijuana Program
PDMP: Prescription Drug Monitoring Program Registry
PMR: physical medicine and rehabilitation
THC: tetrahydrocannabinol
UDS: urine drug screen
