# Supplementary material for: New York Physicians' Perspectives and Knowledge of the State Medical Marijuana Program
Source: Cannabis Cannabinoid Res. 2018 Mar 1;3(1):74–84. doi: 10.1089/can.2017.0046 (PMC5899285; doi:10.1089/can.2017.0046)
Supplement: Supplemental data [file Supp_Data.pdf]

Supplementary Data

Survey

What is your gender?
- o Male
- o Female
- o Other
- o Prefer not to answer

What is your age?
- o 25 to 34
- o 35 to 44
- o 45 to 54
- o 55 to 64
- o 65 to 74
- o 75 or older

What is your professional degree? (Check all that apply)
- o MD
- o DO
- o Other (please specify; i.e. PhD, JD, MBA, MSPH, etc.)

How many years are you in practice?
- o <1
- o 1–5
- o 6–10
- o 11–15
- o 16–20
- o 21–25
- o >25

In which NY County do you practice?
- o Prefer not to answer
- o Please specify:

What is your specialty?
- o Prefer not to answer
- o Please specify:

What is your practice setting?
- o Academic/University Hospital
- o Public Hospital (non-VA)
- o Private Practice
- o VA Hospital

- o Prefer not to answer
- o Other (please specify)

How would you rate your knowledge of the endocannabinoid system? (1 = uninformed; 5 = very well informed)

O 1 O 2 O 3 O 4 O 5

Please rate the following statement: "Medical Marijuana (MMJ) should be an option available to patients."
- o Strongly agree
- o Somewhat agree
- o Neutral
- o Somewhat disagree
- o Strongly disagree

Are you familiar with the requirements for a patient to be able to obtain MMJ in NY?
- o Yes
- o No
- o Somewhat familiar

Are you familiar with the requirements for a physician to be able to certify a patient for MMJ in NY?
- o Yes
- o No
- o Somewhat familiar

What do you think of the number of conditions and symptoms that qualify for MMJ certification in NY?
- o Enough
- o Too many
- o Too few
- o I am not sure

What do you think of the current MMJ formulations and routes of administration approved in NY?
- o Enough
- o Too many
- o Too few
- o I am not sure

Have you registered with NY State to certify patients for MMJ?
- o Yes
- o No
- o Prefer not to answer

Which factors influenced your decision to register or not? Please mark all that apply:
- □ My medical specialty
- □ Experiences with patients
- □ Medical Literature
- □ Other Physicians/Colleagues
- □ News/media
- □ Lectures/CME
- □ Friend/family use
- □ Practice/Workplace policy

- ☐ Federal Law/Marijuana is a Schedule I drug
- ☐ Other (please specify)

Have your patients inquired about MMJ within the past year?

- o Yes
- o No

Are you willing to discuss MMJ with your patients?

- ☐ Yes, but only if my patient expresses interest first
- ☐ Yes, if I feel that my patient would benefit
- ☐ Yes, I am willing to discuss but I would not recommend its use under any circumstances
- ☐ No, I am not willing to discuss
- ☐ Not applicable
- ☐ Other (please specify):

Have any of your patients used marijuana (illicit of medical) for symptom control?

- o Yes
- o No
- o I don't know

If yes, for what symptoms? Please mark all that apply:

- ☐ Pain
- ☐ Cachexia
- ☐ Nausea
- ☐ Depression
- ☐ Anxiety
- ☐ Other (please specify)

Have you prescribed FDA-approved cannabinoids (Dronabinol or Nabilone) for your patients?

- o Yes
- o No

If you would consider cannabinoids in your treatment plan, would you first opt for FDA-approved cannabinoids or NYS-approved MMJ formulations?

- o FDA-approved cannabinoids
- o NYS-approved MMJ formulations
- o Not applicable

(OPTIONAL) Please explain your choice:

Do you prescribe opioids to your patients?

- o Yes
- o No
- o Prefer not to answer

In general, which do you feel has a greater risk profile?

- o MMJ
- o Opioids
- o I am not sure
