# Supplementary material for: New York Physicians' Perspectives and Knowledge of the State Medical Marijuana Program
Source: Cannabis Cannabinoid Res. 2018 Mar 1;3(1):74–84. doi: 10.1089/can.2017.0046 (PMC5899285; doi:10.1089/can.2017.0046)
Supplement: Supplemental data [file Supp_Table1.pdf]

Supplementary Table S1. Societies, Academic Departments and Organizations Contacted to Participate in Survey

Specialty societies
New York Society of Addiction Medicine
The NYS Society of Allergy & Immunology, Inc.
NYS Society of Anesthesiologists, Inc.
NYS Chapter of the American College of Cardiology
NYS Society of Dermatology and Dermatologic Surgery
NYS Chapter American College of Emergency Physicians
NYS Academy of Family Physicians
New York Chapter American College of Physicians
New York State Neurological Society, Inc.
NYS Neurosurgical Society, Inc.
American Congress Obstetricians & Gynecologists-District II
New York Occupational and Environmental Medical Association
NYS Society of Medical Oncologists and Hematologists, Inc.
NYS Ophthalmological Society
NYS Society of Orthopedic Surgeons, Inc.
NYS Society of Otolaryngology–Head and Neck Surgery
NYS Society of Pathologists
American Academy of Pediatrics- District II
NYS Society of Physical Medicine and Rehabilitation, Inc.
NY Regional Society of Plastic and Reconstructive Surgery
New York State Society of Plastic Surgeons
NYS Psychiatric Association, Inc.
The NYS Radiological Society, Inc.
New York State Rheumatology Society
NYS Chapter of the American Society for Metabolic and Bariatric Surgery
New York Chapter of the American College of Surgeons, Inc.
NY Society for Surgery of the Hand
New York State Thoracic Society
NYS Urological Society
New York Society of Interventional Pain Physicians
NY Pain Society
NYS of Osteopathic Medicine
New York Academy of Medicine
Medical Society of the State of New York
County societies
Medical Society County of Albany
Medical Society County of Allegany
Bronx County Medical Society, Inc.
Broome County Medical Society, Inc.
Medical Society County of Cattaraugus
Cayuga County Medical Society, Inc.
Medical Society County of Chautauqua
Chemung County Medical Society, Inc.
Medical Society County of Chenango
Medical Society County of Clinton
Medical Society of the County of Columbia, Inc.
Cortland County Medical Society
Delaware County Medical Society
Dutchess County Medical Society
Medical Society County of Erie
Medical Society County of Essex
Medical Society of the County of Franklin, Inc.
Medical Society of the County of Fulton
Medical Society County of Genesee
Medical Society County of Greene
Medical Society County of Herkimer
Jefferson County Medical Society
Medical Society County of Kings, Inc.
Lewis County Medical Society
Medical Society of Livingston, Inc.
Medical Society County of Madison, Inc.
Monroe County Medical Society
Medical Society of the County of Montgomery
Nassau County Medical Society, Inc.
New York County Medical Society, Inc.
Medical Society County of Niagara
Medical Society County of Oneida, Inc.
Onondaga County Medical Society, Inc.
Medical Society of the County of Ontario, Inc.
Medical Society County of Orange, Inc.
Medical Society County of Orleans
Medical Society County of Oswego
Otsego County Medical Society
Putnam County Medical Society
Medical Society County of Queens, Inc.
Medical Society County of Rensselaer
Richmond County Medical Society, Inc.
Medical Society County of Rockland
Saratoga County Medical Society, Inc.
Medical Society County of Schenectady
Medical Society of the County of Schoharie
Schuyler County Medical Society
Seneca County Medical Society, Inc.
Medical Society of the County of St. Lawrence, Inc.
Steuben County Medical Society, Inc.
Suffolk County Medical Society, Inc.
Medical Society County of Sullivan
Tompkins County Medical Society
Medical Society County of Ulster
Warren County Medical Society
Medical Society County of Washington
Wayne County Medical Society
Medical Society County of Westchester
Medical Society of the County of Wyoming
Medical Society of the County of Yates
Academic departments in medical centers
NYU School of Medicine
Icahn School of Medicine Mount Sinai
New York Presbyterian Weill Cornell Medical Center
New York Presbyterian Columbia University Medical Center
New York Medical College
Montefiore Medical Center
State University of New York Upstate
Memorial Sloan Kettering Cancer Center
State University of New York Downstate
State University of New York Stony Brook
New York Institute of Technology
Northwell/Hofstra
SUNY Upstate Medical University
Others
NYC Health Commissioner
Iroquois Healthcare Association
Independent Doctors of NY
Public Health Association of NYC
Compassionate Care NY

(*continued*)

Supplementary Table S1. **(Continued)**
