# Supplementary material for: New York Physicians' Perspectives and Knowledge of the State Medical Marijuana Program
Source: Cannabis Cannabinoid Res. 2018 Mar 1;3(1):74–84. doi: 10.1089/can.2017.0046 (PMC5899285; doi:10.1089/can.2017.0046)
Supplement: Supplemental data [file Supp_Table2.pdf]

Supplementary Table S2. Additional Reasons Influencing Decision Not to Register

Other reasons influencing decision NOT to register
Not enough evidence ($n = 10$)
There is no medical indication. If a medical indication can be clearly indicated, then the compound needs to be approved and followed by FDA
Lack of scientific knowledge on how it works and who should use it
Not enough data to say yes
Lack of studies and clarity of policies
In my field, there is very poor evidence base to support MMJ
Mixed outcomes evidence and variable products + weak studies
No evidence to support use (except possibly pediatric seizures)
No medical indication for "pot" exists; THC is already available for many years. The Netherlands has extensive experience with recreational use of "pot" and physicians there never prescribe it
It is currently not an appropriate therapeutic option
I remain unconvinced of the value
Time-consuming, burdensome process ($n = 8$)
Not practical or affordable, therefore unrealistic
The practical logistics/requirements of registering/maintaining are extremely burdensome
Barriers to apply
No more time in my life!
I need to find time to finish the registration process
Too difficult to deal with
Minimal likelihood of use; process too complicated
I took the medical marijuana webinar preparation, but then found out it cost $5000 to begin
Not relevant to practice ($n = 7$)
Not really in my practice (×5)
I don't prescribe medications, other than postoperative medicines
My patients don't need it
Lack of Knowledge ($n = 7$)
Lack of knowledge
Minimal knowledge
I know nothing about it
I wasn't aware of the option
Not familiar enough yet
Do not know how
How to apply is unclear
Don't believe in legitimacy of MMJ ($n = 5$)
Cultural bias against it from 20th Century
Dislike of use of marijuana
Don't believe in using marijuana
Medical Marijuana is in the same health category as medical alcohol
'Medical' marijuana is the way entrepreneurs are using to bring recreational marijuana to market, like physicians advertising cigarettes in the 1950s
Practice Considerations ($n = 4$)
Patients would beat down my door and malinger
I do not wish to receive referrals for this specific issue
Did not want to be on a registry where this right would then become a focus of the practice
My patient cohort is one in which the advantages and disadvantages of MMJ are particularly challenging to sort through, and my employer is partially federally funded. Loss of federal funding would be a very bad thing
Safety Issues ($n = 3$)
Harmful effects of marijuana, especially on psychiatric illness onset and exacerbation
There is more harm than benefit for patients
Other medications better meet treatment goals with less side effects and fewer adverse issues
Legal ramifications ($n = 2$)
I think writing Rxs opens the physician up for unwanted lawsuits
NY marijuana law criminalizes off indication prescribing as felony
Marijuana should be legal for recreational use ($n = 1$)
Have not thought about it ($n = 1$)

Open-ended responses were thematically categorized and quantified.

FDA, Food and Drug Administration; MMJ, medical marijuana; THC, tetrahydrocannabinol.
